# Correction to: COVID-19 in pediatric cancer patients is associated with treatment interruptions but not with short-term mortality: a Polish national study

**DOI:** 10.1186/s13045-022-01279-3

**Published:** 2022-05-31

**Authors:** Jadwiga Węcławek-Tompol, Zuzanna Zakrzewska, Olga Gryniewicz-Kwiatkowska, Filip Pierlejewski, Ewa Bień, Agnieszka Zaucha-Prażmo, Olga Zając-Spychała, Anna Szmydki-Baran, Agnieszka Mizia-Malarz, Wioletta Bal, Małgorzata Sawicka-Żukowska, Agnieszka Kruk, Tomasz Ociepa, Anna Raciborska, Agnieszka Książek, Tomasz Szczepański, Jarosław Peregud-Pogorzelski, Maryna Krawczuk-Rybak, Radosław Chaber, Michał Matysiak, Jacek Wachowiak, Ninela Irga-Jaworska, Wojciech Młynarski, Bożenna Dembowska-Bagińska, Walentyna Balwierz, Agnieszka Matkowska-Kocjan, Bernarda Kazanowska, Jan Styczyński, Marek Ussowicz

**Affiliations:** 1 Department and Clinic of Pediatric Oncology, Haematology and Bone Marrow Transplantation, Wroclaw Medical University, Borowska 213, 50-556 Wroclaw, Poland; 2 Department of Pediatric Oncology and Hematology, Institute of Pediatrics, Jagiellonian University, Collegium Medicum, Kraków, Poland; 3 Department of Pediatric Oncology, Children’s Memorial Health Institute, Warsaw, Poland; 4 Department of Pediatrics, Hematology and Oncology, Medical University of Lodz, Lodz, Poland; 5 Department of Pediatrics, Hematology and Oncology, Medical University of Gdansk, Gdansk, Poland; 6 Department of Pediatric Hematology, Oncology and Transplantology, Medical University of Lublin, Lublin, Poland; 7 Department of Pediatric Oncology, Hematology and Transplantology, University of Medical Sciences, Poznan, Poland; 8 Department of Oncology, Pediatric Hematology, Transplantology and Pediatrics, Children’s Hospital, Medical University of Warsaw, Warsaw, Poland; 9 Department of Pediatrics, Medical University of Silesia, Katowice, Poland; 10 Clinic of Pediatric Oncology and Hematology, State Hospital 2 in Rzeszow, Rzeszow, Poland; 11 Department of Paediatrics, Institute of Medical Sciences, University of Rzeszow, Rzeszow, Poland; 12 Department of Pediatric Oncology and Hematology, Medical University of Bialystok, Białystok, Poland; 13 Department of Paediatrics, Paediatric Oncology and Immunology, Pomeranian Medical University, Szczecin, Poland; 14 Department of Oncology and Surgical Oncology for Children and Youth, Institute of Mother and Child, Warsaw, Poland; 15 Department of Pediatric Hematology and Oncology, Zabrze, Medical University of Silesia, Katowice, Poland; 16 Department and Clinic of Pediatric Infectious Diseases, Wroclaw Medical University, Wroclaw, Poland; 17 Department of Pediatric Hematology and Oncology, Jurasz University Hospital, Collegium Medicum Nicolaus Copernicus University Torun, Bydgoszcz, Poland; 18 Department of Pediatrics, Hemato-Oncology and Gastroenterology, Pomeranian Medical University, Szczecin, Poland

## Correction to: J Hematol Oncol (2021) 14:163 10.1186/s13045-021-01181-4

The original article [1] mistakenly omitted two co-authors—Tomasz Ociepa and Ninela Irga-Jaworska—who have both since been re-instated into the authorship.
